# Parkinson's Impulse‐Control Scale for the Severity Rating of Impulse‐Control Behaviors in Parkinson's Disease: A Semistructured Clinical Assessment Tool

**DOI:** 10.1002/mdc3.12316

**Published:** 2016-01-25

**Authors:** David Okai, Sally Askey‐Jones, Joel Mack, Anne Martin, Kallol Ray Chaudhuri, Michael Samuel, Anthony S. David, Richard G. Brown

**Affiliations:** ^1^ Section of Cognitive Neuropsychiatry Institute of Psychiatry, Psychology, and Neuroscience King's College London London United Kingdom; ^2^ Department of Psychological Medicine Oxford University Hospitals NHS Trust Oxford United Kingdom; ^3^ School of Psychology Newcastle University Newcastle upon Tyne United Kingdom; ^4^ Portland Veterans Affairs Medical Center Portland Oregon USA; ^5^ King's Health Partners King's College Hospital NHS Trust London United Kingdom; ^6^ Department of Psychology Institute of Psychiatry, Psychology and Neuroscience King's College London London United Kingdom

**Keywords:** impulse‐control disorder, Parkinson's disease, severity, rating, carer

## Abstract

**Background:**

Impulse‐control behaviors (ICBs) are increasingly recognized in Parkinson's disease (PD) as drug‐related effects of dopaminergic mediation that occur in 15% to 35% of patients with PD. The authors describe the design and evaluation of a new, clinician‐rated severity scale for the assessment of syndromal and subsyndromal forms of impulse‐control disorders (ICDs), simple (punding) and complex (hobbyism) repetitive behaviors, and compulsive overuse of medication (dopamine dysregulation syndrome).

**Methods:**

The Parkinson's Impulse‐Control Scale (PICS), the first PD‐specific, semistructured interview to cover the full range of PD‐related ICBs, is described along with initial evidence on its clinimetric properties including interrater reliability, discriminant validity and sensitivity to change. A convenience sample of PD patients with ICBs and those without were administered a semistructured interview (n = 92).

**Results:**

The scale distinguished between those with and without clinically detected ICBs and between patients with syndromal ICD and subsyndromal ICB (receiver operating characteristic areas under the curve, 92%–95%). Cutoff values were suggested, and substantial agreement was reported on weighted kappa (Κ) values for clinician‐clinician rating of severity (Κ = 0.92). Significant improvements were detected on the scale after a randomized controlled trial of cognitive‐behavioral therapy and medication adjustment (*t*[22] = 5.47; *P* < 0.001).

**Conclusions:**

The PICS appears to be a reliable measure of the full range of PD ICBs with good levels of interrater reliability. It may provide a useful measure to assess the severity of ICBs and monitor change in clinical and research settings; although, given the specialized centers used for recruitment of this sample, further psychometric evaluation is required.

Impulse‐control disorder (ICD) in Parkinson's disease (PD) describes problematic behaviors that include pathological gambling, compulsive shopping, compulsive eating, altered sexual behavior, hobbyism, punding, and dopamine medication overuse (dopamine dysregulation syndrome [DDS]).^1^, ^2^, ^3^ Such problems occur in 15% to 35% of people with PD^4^, ^5^ and are variably associated with insight.^6^ Where they impact significantly on social and occupational functioning, they warrant the term “disorder,” but they lie on a continuum of severity, suggesting that a dimensional approach to the assessment of impulse‐control behaviors (ICBs) may be most appropriate.^2^

A variety of generic and PD‐specific ICD/ICB screening tools exist.^7^, ^8^, ^9^, ^10^ The Questionnaire for Impulsive‐Compulsive Disorders in Parkinson's Disease (QUIP) is the most commonly used, validated, self‐report screening tool and assesses the ICDs listed above.^11^ A corresponding rating scale (QUIP‐RS) is available to rate the severity of the same behaviors and provide a measure of change over time.^12^

Self‐report scales have considerable utility in research and clinical practice but may have limited usefulness where the individual lacks insight or seeks to minimize aspects of the behavior.^13^ Clinician‐rated instruments based on semistructured interviews with operationalized scoring criteria can provide a more detailed quantitative assessment in some settings. Such an assessment can also aid in the clinical decision regarding whether the behaviors have an impact on social and occupational functioning sufficient for caseness as a *Diagnostic and Statistical Manual of Mental Disorders, fourth edition* (DSM‐IV) (or DSM‐aligned) disorder. To our knowledge, there are no PD‐specific, semistructured clinical assessment tools designed to cover all of the most common ICBs.

Recently, we published results from a randomized controlled trial of a cognitive‐behavioral intervention for the management of ICBs in PD.^14^, ^15^ A new clinician‐rated scale—the Parkinson's Impulse‐Control Scale (PICS) (formerly known as the Impulse‐Control Behavior Severity Scale)—was developed for the trial and was used as a secondary outcome. The QUIP‐RS was not available at the time. The PICS was found to be sensitive to change in ICB severity resulting from treatment. Here, we report on the scales' validity, reliability, and sensitivity to change after routine medical management (medication adjustment) and the above‐mentioned cognitive‐behavioral therapy (CBT) intervention.

## Materials and Methods

### Scale Description

#### PICS

The PICS is a clinician‐rated scale based on a semistructured interview that measures both the intensity of each ICB (indicated by frequency and scale of the behavior) and its individual and social impact, which are combined to provide an index of severity. It covers 7 ICBs; gambling, shopping, eating, hypersexuality, simple (punding) and complex (hobbyism) repetitive behaviors, and compulsive overuse of medication (DDS).^2^, ^4^

For illustration, the questions and scoring criteria for the Gambling Behavior scale of the PICS are provided online (see online supporting information). A copy of the full PICS is available from the authors on request. Each ICB subscale comprises an initial 3‐item screening questionnaire (yes/no responses) to determine: (1) the presence of any ICBs in the preceding month; (2) the relation between any behavior and PD; and (3) whether they, or their carer/partner, believe that the behavior has worsened since starting medications (potentially minimizing underreporting/denial of symptoms).

These screening questions were developed based on a focus group, a review of existing scales (Evans et al., unpublished results), and discussions with experts who had an interest in ICB. The timeframe of 1 month was selected as sufficient to detect infrequent ICBs while still allowing the scale to measure change. The use of screening questions was designed to save time, because no ICB or preexisting ICB that was not plausibly linked to PD or medication use would not be further assessed. If, on the basis of this information, the clinician deems the behavior to be present and PD‐related, then further questions are asked for that ICB. Structured questions specific to the behavior are used to elicit information related to its intensity and impact on the individual and others. This quantitative and qualitative information is recorded but not scored. Rather, the information, combined with that from other sources (e.g, collateral history), informs the final clinical rating of behavior on an operationalized scale from 1 to 4 for intensity and from 1 to 3 for impact. A score of 0 is assigned when the ICB is absent. For each ICB, the clinician‐rated intensity and impact scores are multiplied to give a single severity score between 0 and 12. Finally, the clinician is able to indicate their confidence based on the information available. A total severity score can be derived as the sum of the 7 ICBs with a range from 0 to 84 (higher scores denote greater severity).

When eliciting information, the interview enquires about the behavior both “on average” and at its maximum. These extremes, even if infrequent, are important in forming clinical judgements about intensity and impact. For example, gambling losses may be typically modest but large on occasions. Ratings also take into account the individual context of the behavior, particularly for impact. For example, an individual may place frequent bets but have a large income. Therefore, the behavior will be seen as having limited impact but will be important for meaningful clinical interpretation.

The screening questions can be completed in less than 2 minutes if there are no ICBs. For those with 2 or 3 ICBs, the scale typically can be completed in 5 to 10 minutes. Administration and scoring require a degree of training or clinical familiarity with ICBs in PD.

#### Participants

In total, 92 patients with PD were interviewed face‐to‐face or by phone (Table 1). Patients with and without known ICBs were purposely sampled from a variety of sources to provide a group with problems spanning a wide range of severities. We assessed the eligibility of 45 patients who had clinically evident ICBs for possible inclusion in our randomized controlled trial.^14^ An additional 41 participants were recruited as a convenience sample from King's College Hospital Movement Disorder Service and comprised patients with known ICBs as well as patients thought to have no ICB. A further 6 participants responded to an advert posted on Parkinsons.org.uk (British‐based) and PatientsLikeMe.com (US‐based). One author (D.O.) performed baseline assessments on all patients and repeat measures at 6 months. An additional 12 patients were independently assessed by another author (J.M.) for levels of agreement. Patients with ICBs who had not had a trial of dopamine agonist reduction were reassessed at 6 months after an attempted gradual tapering of medication by their treating clinician.

**Table 1:** Demographic and Clinical Information (N = 92)

	Mean ± SD or No. of Patients (%)	
	ICB Group, n = 67	Non‐ICB Group, n = 25
Patient Characteristics		
Age, y	59.6 ± 8.7	62.5 ± 10.9
Men	46 (69)	17 (68)
Duration of PD, y	10.2 ± 6.0	9.0 ± 6.3
Duration of ICB, y	4.1 ± 3.8	—
sMMSE total score^a^	28.3 ± 2.2	28.6 ± 1.3
TICS‐M total score: Range, 0–39^b^	21.1 ± 4.2	26.8 ± 2.8
Marital status		
Single	3 (5)	3 (12)
Married/cohabiting	47 (73)	18 (72)
Separated/divorced	6 (8)	3 (12)
Widowed	9 (14)	1 (4)
Ethnic origin		
White	63 (94)	23 (92)
Other	4 (6)	2 (8)
Education^c^		
Left school at age <14 y	6 (10)	0 (0)
Left school at age 14–15 y	21 (33.9)	5 (20)
Left school at age >16 y	35 (57)	20 (80)
Medication		
UPDRS		
UPDRS‐III^d^	29.7 ± 13.9	29 ± 15.5
UPDRS‐IV^e^	7.8 ± 4.4	5.3 ± 5.2
Hoehn and Yahr	2.0 ± 1.2	2.4 ± 1.2

a MMSE: ICB group, n = 58; non‐ICB group, n = 17.

b TICS‐M: ICB group, n = 8; non‐ICB group, n = 6.

c Education: ICB group, n = 62.

d UPDRS: ICB group, n = 53; non‐ICB group, n = 18.

e ICB group, n = 56; non‐ICB group, n = 18.

SD, standard deviation; ICB, impulse‐control behavior; PD, Parkinson's disease; sMMSE, Standardized Mini‐Mental State Examination; TICS‐M, Telephone Interview of Cognitive Status‐Modified; UPDRS, Unified Parkinson's Disease Rating Scale; UPDRS‐III, UPDRS part 3 (clinician‐scored monitored motor evaluation); UPDRS‐IV, UPDRS part 4 (complications of therapy).

Exclusion criteria were Standardized Mini‐Mental State Examination^16^ scores < 24 (for face‐to‐face assessments) or Telephone Interview of Cognitive Status‐Modified^17^ scores < 19 (for telephone assessments). The study was approved by the National Research Ethics Service (ref no: 10/H0716/46). Informed consent was obtained from both patients and caregivers.

#### Clinimetric Assessment

Data quality was assessed (including missing data), and a 5% value for noncomputable or missing data was deemed acceptable as the limit.^18^ The purposeful (nonrandom) nature of the sample selection meant that estimates of scale‐skewness and floor and ceiling effects could not be meaningfully computed.^19^ Acceptability of the scale was judged on the basis of participant willingness to complete the assessment and answer all questions.

Discriminant validity was assessed by comparing PICS scores between patients with and without evidence of a current or recent ICB. Presence of an ICB was determined based on the QUIP^11^ and additional questions to assess DSM‐IV criteria for pathological gambling, binge eating disorder, Voon's criteria for hypersexuality, and McElroy's criteria for shopping.^8^, ^20^, ^21^

For patients who had an ICB, we assessed the ability of the PICS to discriminate between those who met criteria for a syndromal ICD and those who did not. This assessment was restricted to eating, sex, gambling, and shopping where operationalized diagnostic criteria exist. Symptoms that did not meet full diagnostic criteria for each of the disorders were labeled as subsyndromal; then, the ability of the PICS to discriminate validity was assessed for each behavior/disorder individually. A receiver operating characteristics analysis provided the area under the curve and optimal cutoff scores to determine syndromal disorder.

Responsiveness to change was assessed in 2 subgroups using a paired *t* test for each sample. The first was the total sample of patients (N = 41) who completed CBT for the management of their ICB. Patients were assessed before and at the end of treatment (fixed time point, 6 months). The second subgroup was a sample of patients (N = 23) identified with an incident ICB who were then managed medically by adjustment of their antiparkinsonian medication. Patients were assessed before adjustment and after 6 months.

A limited assessment of interrater reliability was based on 12 patients who were receiving stable treatment by independent ratings approximately 1 month apart. These assessments were performed by 2 authors (J.M. and D.O.). Test‐retest reliability was assessed on the subsample of CBT waitlist control patients (n = 17) who were receiving stable antiparkinsonian treatment 6 months after initial assessment. Quadratic weighted kappa (Κ) values were used for both measures of reliability.

## Results

All interviews were successfully completed in full with no refusals to answer questions or other sources of missing data. Baseline characteristics are presented in Table 1. There were no significant differences between groups based on demographic or clinical characteristics. Of the 92 patients assessed, 28 (25%) screened positive for 1 ICD, 18 (16%) screened positive for 2 ICDs, and 9 (8%) screened positive for 3 ICDs. Six patients had hobbyism, punding, or DDS. ICBs were further identified in 4 patients who were referred by clinicians as part of the non‐ICB convenience sample (all had hypersexuality).

The frequencies of ICBs and PICS subscores for those who scored positive and negative on the QUIP are reported in Table 2. PICS screening questions confirmed the presence of a QUIP‐indicated ICB in all patients and identified an additional 19 patients who had eating, sex, gambling, and DDS ICBs.

**Table 2:** Parkinson's Impulse‐Control Scale Scores by Problem for Participants Scoring >0 on Each Subscale

Variable	No. (%)^a^	PICS Subscale Score: Mean ± SD	QUIP No. Positive:Negative	Syndromal ICB: No. (%)^a^	Mean ± SD Score [Scoring Range]	
					Syndromal	Subsyndromal
Eating	31 (26)	3.4 ± 2.5	21:10	14 (45)	5.7 ± 2.1 [4–12]	1.4 ± 0.6 [1–3]
Sex	28 (24)	6.4 ± 4.1	24: 4	21 (75)	8.4 ± 3.4 [3–12]	1.9 ± 0.7 [1–3]
Gambling	16 (14)	5.5 ± 3.1	14: 2	12 (75)	6.8 ± 2.5 [4–12]	1.8 ± 1.0 [1–3]
Shopping	16 (14)	3.8 ± 2.2	16:0	8 (50)	5.4 ± 2.0 [3–9]	2.3 ± 1.0 [1–4]
Punding	4 (3)	5.3 ± 2.5	4:0	NA	—	—
Hobbyism	8 (10)	5.5 ± 1.9	8:0	NA	—	—
DDS/*off* period dysphoria	9 (8)	6.4 ± 2.1	6:3	NA	—	—
Total	112	4.9 ± 3.2	81:19	56 (62)	6.6 ± 3.0 [3–12]	1.7 ± 0.8 [1–4]

a Values are shown as the percentage of those who had 1 or more impulse‐control behavior(s).

SD, standard deviation; PICS, Parkinson's Impulse‐Control Scale; QUIP, Questionnaire for Impulsive‐Compulsive Disorders in Parkinson's Disease; ICB, impulse‐control behaviors; NA, not accessible; DDS, dopamine dysregulation syndrome.

Interviewer confidence in accuracy of the ratings for the scale was “acceptable” in 59%, defined as probably reflecting the approximate nature and scale of the problem; and “good” in 41%, defined as likely to reflect the true nature and scale of the problem (usually based on informant corroboration).

### Discriminant Validity

The mean ± standard deviation (SD) total PICS score for those who screened positive for 1 or more ICBs (N = 67) was 8.9 ± 5.7 (range, 1–26) compared with 0 ± 0 for those without an ICB (N = 25). Further analysis was restricted to those patients who screened positive for an ICB on the PICS.

Mean ± SD PICS scores across the 4 index ICBs were significantly higher for those who met the criteria for syndromal ICD (6.6 ± 3.0; range, 3–12) compared with those who had subsyndromal ICD (1.7 ± 0.8; range, 1–4; *t*[90] = 9.4; *P* < 0.05). Receiver operating characteristics analysis showed high areas under the curve from 92% to 95% (Table 3).

**Table 3:** Discriminant Validity of the Parkinson's Impulse‐Control Scale (N = 92)^a^

	Receiver Operating Characteristics								
Subscale	≥1	>1	>2	>3	>4	>6	>8	>9	>12
Eating (AUC = 0.981)									
Sensitivity, %	100	100	100	93^b^	73	20	13	—	0
Specificity, %	0	56	89	89	100	100	100	—	100
Sex (AUC = 0.928)									
Sensitivity, %	100	95	91^b^	73	—	59	—	27	0
Specificity, %	0	33	83	100	—	100	—	100	100
Gambling (AUC = 0.982)									
Sensitivity,%	100	100	100	100	82^b^	36	—	9	0
Specificity, %	0	40	60	80	100	100	—	100	100
Shopping (AUC = 0.944)									
Sensitivity, %	100	100	100	80^b^	70	30	20	10	0
Specificity, %	0	25	63	87.5	100	100	100	100	100

a The table shows area under the curve (AUC) values for each subscale.

b The optimal cutoff with both sensitivity and specificity ≥80% for each subscale.

### Responsiveness to Change

A subset of 23 patients was examined before and after adjustment (typically reduction) of the antiparkinsonian medication. Dopamine agonist was discontinued in 40% of patients. The initial mean ± SD levodopa‐equivalent dose (LEDD) was 1207.8 ± 612.6 mg, with reduction to a mean of 922.6 ± 525.2 mg (the dopamine agonist LEDD was reduced from 214.6 ± 38.4 mg to 142.8 ± 127.9 mg). The mean ± SD total PICS score fell from 10.4 ± 5.8 before dose reduction to 3.1 ± 3.1 after reduction (*t*[22] = 5.47; *P* < 0.001; effect size, 1.6). In addition, the scale proved sensitive to change in those who underwent treatment in the context of a psychosocial intervention as reported elsewhere.^14^

### Interrater and Test‐retest Reliability

Agreement for interrater reliability and for test‐retest is shown in Table 4. The 2 clinicians showed substantial agreement on the 12 participants assessed. For those with ICBs who were on the waiting list for the psychosocial intervention (n = 17), PICS total scores at baseline and at 26 weeks were moderately associated with baseline scores. Mean scores ± SD for individual ICBs were 4.50 ± 2.9 at baseline and 3.68 ± 2.8 at 6 months (*t*[16] = 1.13; *P* = 0.27).

**Table 4:** Interrater Agreement and Convergent Report on the Parkinson's Impulse‐Control Scale^a^

	No.	Weighted Κ	SE	95% CI
Interrater	12	0.92	0.03	0.86–0.97
Waitlist at 6‐mo to 12‐mo follow‐up	17	0.42	0.2	0.01–0.83

a Scores of 0.01–0.20 indicate slight agreement; 0.21–0.40, fair agreement; 0.41–0.60, moderate agreement; 0.61–0.80, substantial agreement; 0.81–1.00, almost perfect agreement.

SE, standard error; CI, confidence interval.

## Discussion

The PICS is a brief, clinician‐rated screening and severity tool and provides a means of gathering comprehensive data, allowing an assessment of the intensity and impact of a wide range of ICBs common in PD. The preliminary results presented suggest that the scale is practical to use, acceptable to patients, and demonstrates a degree of reliability and sensitivity to change.

The PICS offers an alternative or adjunct to the recently published QUIP‐RS,^12^ from which it differs in a numbers of respects. The mode of administration of the scale (self‐report or clinician‐rated) is important, with each method conferring different advantages: economy for the QUIP‐RS and the ability to make clinically informed ratings for the PICS, but with a cost to time and the need for an experienced rater. The QUIP‐RS also focusses attention to those behaviors that the patient identifies as problematic and uses frequency as the main indicator or severity. This risks missing instances in which a patient minimizes the impact of an ICB or underestimates a behavior that is relatively infrequent but intense. For the PICS, observation of behavior is recorded regardless of whether it is believed to be a problem, and this is followed by an exploration of typical and extreme variation in behavior to make a clinically informed decision. It can also be combined with other sources when making overall ratings. Similarly, the PICS can better determine, from the outset, whether the ICB may be a preexisting condition independent of PD or whether it has emerged or worsened with PD and its treatment. Ultimately, choice between the 2 scales in research and clinical practice will depend on the purpose of the assessment. The 2 forms of administration may suggest the use of both measures to provide complementary evidence on severity.

This study offers preliminary cutoffs for the PICS subscales for identifying syndromal forms of the behaviors with high levels of specificity, but these will need confirmation with larger samples. The high positive, and low negative likelihood ratios in each ICBs signify the respective probabilities of having or not having the disorder at a clinical cutoff point. Optimal cutoffs for scales where a gold‐standard syndromal diagnosis is not available remain to be determined but a figure of 4 or 5 is suggested as appropriate in the first instance. The PICS and subscales also proved sensitive to change in those who had a reduction in their medication. This is the usual clinical approach to management of this range of conditions and hence the scale may have utility in day‐to‐day clinical monitoring of ICBs and ICDs. The identification of previously clinically unrecognized ICBs suggests that the systematic format of the interview is acceptable to patients who report behaviors judged (clinically) to indicate an ICB.

In relation to DDS, the scale takes a broad approach to identification and assessment and thus may produce different estimates of prevalence and severity than other measures. Our conceptualization of DDS includes not only the small proportion of individuals who medicate to seek a medication high^3^ but also those who develop a phobic or anticipatory avoidance of being *off*, losing control of their daily regimen, with “rescue” medications to deleterious effect.^2^

The preliminary interrater reliability results suggest that the ratings for intensity and impact allow agreement when descriptions of ICBs are sufficiently operationalized. The lower test‐retest reliability may indicate a limitation of the scale but could also reflect true clinical variation in the severity of the behaviors during the interval.

Nonetheless, the development of the scale has several limitations. Many of the diagnostic criteria for the PD ICBs in themselves lack validation, whereas we lack operationalized criteria for punding, hobbyism, and DDS. As such, the focus of the present study was not on validation of the tool for screening of ICDs (the screening questions were used to reduce the time burden of the assessment). In addition, the scale may need to be adapted for use outside the United Kingdom, particularly where population rates and cultural norms surrounding the behaviors may differ. As with many multidimensional scale measures of both frequency and intensity of a behavior, a patient with 1 very severe ICD theoretically could score the same as someone with 2 or 3 milder ICDs (not affecting function). In practice, we observed quite the opposite: the patients tended to have 1 dominant ICB or several minor ICBs that scored a low aggregate, which was supported by our findings on mean scores for syndromal and subsyndromal ICBs. Finally, given the unblinded nature of the assessments and the purposeful sampling of ICBs, caution is necessary in relation to sensitivity to change.

Subject to further use and confirmation of its metric properties, we suggest that the scale has a role in the assessment of ICBs in clinical and research settings. Further work will be required to evaluate the scale with a variety of raters who are less familiar with the scale in relation to the QUIP‐RS and in nontertiary‐level settings.

## Author Roles

1. Research Project: A. Conception, B. Organization, C. Execution; 2. Statistical Analysis: A. Design, B. Execution, C. Review and Critique; 3. Manuscript Preparation: A. Writing the First Draft, B. Review and Critique.

D.O.: 1A, 1B, 1C, 2A, 2B, 3A

S.A.‐J.: 1A, 1B, 1C, 3B

J.M.: 1B, 1C, 3A, 3B

K.R.C.: 1C, 2A, 2C, 3A, 3B

M.S.: 1A, 1C, 3B

A.S.D.: 1A, 1B, 1C, 2A, 2C, 3A, 3B

R.G.B.: 1A, 1B, 1C, 2A, 2C, 3A, 3B

## Disclosures

**Funding Sources and Conflicts of Interest:** This project was funded by Parkinson's UK. Richard G. Brown, Kallol Ray Chaudhuri and Anthony S. David receive salary support from the National Institute for Health Research (NIHR) Mental Health Biomedical Research Center and Dementia Research Unit at South London and Maudsley National Health Service (NHS) Foundation Trust and King's College London. The views expressed are those of the authors and not necessarily those of the NHS, the NIHR, or the Department of Health. The remaining authors report no sources of funding and no conflicts of interest.

**Financial Disclosures for the previous 12 months:** Michael Samuel has received honoraria for lectures/educational material from UCB and Medtronic; unrestricted educational grants from Solvay and Ipsen; and funding for educational trips from Ipsen, Medtronic, and UCB. K. Ray Chaudhuri has received funding from Parkinson's UK, the NIHR, the European Commission; has received educational grants from UCB, Britannia, Abbvie, and Medtronic; has received honorarium from UCB, Abbvie, Britannia, US Worldmeds, Mundipharma, Medtronic, Napp, Otsuka Pharmaceuticals, and has acted as a consultant for UCB, Abbvie, Britannia, Medtronic, and Mundipharma. Anthony S. David receives royalties from editorship of *Cognitive Neuropsychiatry*. Richard G. Brown has received travel expenses and honoraria for speaking and educational activities from UCB, Teva, GlaxoSmithKline, and Brittania. David Okai, Sally Askey‐Jones, Anne Martin, and Joel Mack report no disclosures.

## Supporting information


**Data S1:** The Clinical Version of the Parkinson's Impulse‐Control Scale (PICS) for Gambling.
