# Supplementary material for: Parkinson's Impulse‐Control Scale for the Severity Rating of Impulse‐Control Behaviors in Parkinson's Disease: A Semistructured Clinical Assessment Tool
Source: Mov Disord Clin Pract. 2016 Jan 25;3(5):494–9. doi: 10.1002/mdc3.12316 (PMC5053221; doi:10.1002/mdc3.12316)
Supplement: Supplementary file 1 — Data S1: The Clinical Version of the Parkinson's Impulse‐Control Scale (PICS) for Gambling. [file MDC3-3-494-s001.docx]

**Supporting Information: The Clinical Version of the Parkinson’s Impulse Control Scale (PICS) for Gambling**

**Screening questions**

*Over the past month have you gambled or placed a bet? This includes any form of gambling - scratch cards, National Lottery, bingo, slot machines, card games, betting on horse races or football matches.*

0 No (circle) [NB Score 0 even if gambled previously but not in past month]

1 Yes

If ‘Yes’ document which from above ____________________________________ and continue.

*Do you or your partner believe this behaviour has worsened in relation to Parkinson’s disease and associated medications?*

0    No       (circle)

1    Yes If ‘Yes’ continue.

2 Engaged in Gambling behaviour prior to Parkinson’s disease but now worse

*Clinician agree given patient/carer account and what is known from history?*

0    No       (circle)

1    Yes If ‘Yes’ continue.

**Intensity of gambling**

1. *How often would you gamble in an average month? (e.g. over the past 6 months). What is the average number of times you would gamble? What would be the most?* [NB: Include all forms of gambling behaviour]

Average Max

Less than once a month 1 1

Once a month 2 2

1 to 3 times a month 3 3

1 to 3 times a week 4 4

4 to 6 times a week 5 5

Once a day 6 6

1 to 3 times a day 7 7

More than 3 times a day 8 8

1. *How often have you gambled in the past month?* (rate 1-8) ______
2. *How long do you spend gambling on each session in the past month? What is the average? What is the longest?*

Average Max

Less than 5 minutes 1 1

5-10 minutes 2 2

10-20 minutes 3 3

20-30 minutes 4 4

30-60 minutes 5 5

1-2 hours 6 6

2-4 hours 7 7

More than 4 hours 8 8

1. *In the past month, what is the typical size of your bet? What is the average? What is the largest?*

Average Max

≤10p 1 1

10p-19p 2 2

20p-49p 3 3

50p-99p 4 4

£1-£4.99 5 5

£5-£9.99 6 6

£10-£20 7 7

>£20 8 8

1. *In the past month, how many bets of these sizes would you place in a typical session?*

Average Max

1 1 1

2-3 2 2

4-5 3 3

5-10 4 4

11-15 5 5

16-25 6 6

26-50 7 7

>50 8 8

1. *What is the largest single bet you have placed in the past month* £ _________
2. *In the past month, what is the largest amount that you have won in a single session?*

[NB session of gambling, not single bet] £ _________

1. *In the past month, what is the largest amount that you have lost in a single session?*

£ _________

**Impact of gambling**

1. *When you have lost money in the past month, has it affected your ability to do other things that you would like to do, or to pay for essential items? Have you had to cut back your spending on treats? Have you had problems paying for bills or having enough money for food or other essentials?*

0 No impact

1 Slight impact on other discretionary activities

2 Moderate impact on discretionary activities and/or some impact on non-discretionary expenditure.

3 Marked impact on other discretionary activities and/or definite impact on non-discretionary expenditure

1. *In the past month have you borrowed money from a family member or friend in order to gamble? How often? Do they know what the money is for? Have you ever taken money from them without telling, intending to replace it afterwards?*

0 Has not borrowed/taken money

1 Has borrowed occasionally (1-2 times)

2 Borrows money regularly (>2 times in past month) with their knowledge.

3 Has taken money from another person without asking permission, and/or borrows with deception

1. *Are you concerned about your gambling? Do you think it is problem? Are you always open about any losses?*

0 No worry or does not admit to worry. Does not consider it a problem.

1 Slight worry reported or apparent from interview. Does not consider it a problem No debt.

2 Moderate worry and/or considers gambling a problem. May be some debt. May hide some losses.

3 Marked concern. Considers gambling a serious problem. Significant debt. Hides/lies about losses.

1. *Is your gambling a concern for your family or friends? Do they think it is a problem?*

0 Others do not express any concern. Do not think it is a problem.

1 Others express slight concern. Do not think it is a real problem

2 Others express moderate concern and/or consider gambling a problem

3 Others express marked concern. Consider gambling a serious problem.

Gambling Intensity in past month (* High/Low stake value needs to take into account individual circumstances)

1 Infrequent low stake* betting. No High stake* betting. Minimal loss risk.

2 More frequent low stake betting, and/or occasional high stake betting. Moderate loss risk.

3 Very frequent low stake betting and/or frequent high stake betting. High loss risk.

4 Very frequent high stake betting. Very high loss risk.

Gambling Impact in past month

1 No or minimal impact on other activities, or non-discretionary expenditure. No worry or concern expressed by self or others. Gambling within financial means. No debt. No borrowing.

2 Moderate social/financial impact on other areas of expenditure. Some/occasional debt. Has borrowed to fund gambling. Moderate concern expressed by self and/or others. Not fully open about loses.

3 Significant social/financial impact. Significant debt problem. Has stolen or used deception to fund gambling. Hides losses. Marked concern expressed by self and/or others.

Gambling Intensity x Impact Score ___________

[NB Score 0, if no gambling behaviour]

Interviewer confidence in ratings

1. Low confidence in accuracy of ratings. Likely to underestimate scale of true problem.
2. Acceptable confidence in accuracy of ratings. Probably reflects approximate nature and scale of problem.
3. Good confidence in accuracy of ratings. Likely to reflect true nature and scale of problem.

© David A Okai, 2013. Permission is given for use in clinical management and in not-for-profit research. All other users should contact the copyright holder for further information
